# Imminent risk of LVEF decline in asymptomatic patients with primary mitral regurgitation

**DOI:** 10.3389/fcvm.2024.1410859

**Published:** 2024-10-14

**Authors:** Jingyi Zheng, Shao-wei Huang, Mustafa I. Ahmed, Betty Pat, Steven G. Lloyd, Oleg F. Sharifov, Thomas S. Denney, Louis J. Dell’Italia

**Affiliations:** ^1^ Department of Mathematics and Statistics, Auburn University, Auburn, AL, United States; ^2^ Division of Cardiovascular Disease, University of Alabama at Birmingham, Birmingham, AL, United States; ^3^ Research & Development Service, Birmingham Veterans Affairs Health Care System, Birmingham, AL, United States; ^4^ Department of Electrical and Computer Engineering, Samuel Ginn College of Engineering, Auburn University, Auburn, AL, United States

**Keywords:** machine learning, predictive longitudinal modeling, asymptomatic primary mitral regurgitation, cardiac MRI, LVEF decline, LV circumferential strain rate

## Abstract

**Background:**

2020 American College of Cardiology/American Heart Association (ACC/AHA) Guidelines state that the ideal time for mitral valve surgery in primary mitral regurgitation (PMR) is when the LV approaches but has not yet reached echocardiographic LV ejection fraction (EF) < 60% or LV end-systolic dimension (ESD) > 40 mm. However, it is difficult to know the imminent risk of crossing this threshold when the surgical outcome is less optimal.

**Objective:**

Using machine learning and statistical models, we have shown that cardiac magnetic resonance (CMR) LV sphericity index (SI) and LV mid circumferential strain rate (SR_circ_) added to LVEF and LVESD predict LVEF < 50% after mitral valve surgery. Here we test the hypothesis that these CMR features predict LVEF < 60% in asymptomatic PMR patients at 18 months.

**Methods:**

33 asymptomatic PMR patients with moderate to severe mitral regurgitation had CMR with tissue tagging at baseline and every 6 months for 18 months. Two types of models were employed to predict LVEF < 60% at 18 months: a model using CMR features at a single time point (e.g., baseline) and a model utilizing repeated measurements over time.

**Results:**

CMR LVEF decreased below 60% in 13 patients over 18 months. LVEF varied over time with an inverse relation to mean arterial pressure and mean end-systolic wall stress. Random Forest models utilizing LV SI, LV mid SR_circ_, LVESD, and LVEF at a single time point (baseline) had a predictive accuracy of 64%. LV SI, LV mid SR_circ_, LVESD and LVEF at baseline, 6, and 12 months achieved a higher predictive accuracy of 79%, improved sensitivity from 57% to 85% than baseline alone and identified a threshold of CMR LVEF 63%–64% signaling LVEF < 60%.

**Conclusion:**

The variability of LVEF due to blood pressure dependence may require a longitudinal study that incorporates LVEF, LVESD, SR_circ_ at multiple time points to identify the threshold at which LVEF is at risk for decline to less than 60%.

## Introduction

It is well recognized that outcomes remain suboptimal in primary mitral regurgitation (PMR) patients (1). Despite guidelines recommending earlier surgical intervention there is a wide disparity in adoption across centers (2). Furthermore parameters for intervention remain crude; for example, guidelines recommend the 60% left ventricular ejection fraction (LVEF) cutoff, in an era where more refined measures of function and geometry are becoming increasingly available. It is imperative to refine models for earlier intervention given the fact that despite pre-operative LVEF > 60%, approximately 20% of PMR patients develop post-operative LV dysfunction and long term outcomes are poor (3–5). In the evaluation of asymptomatic PMR patients with LVEF > 60%, it is difficult to know the ***imminent risk*** of LVEF < 60% in the ensuing 6-18 months.

According to 2020 ACC/AHA Guidelines the ideal time for mitral valve surgery is when the LV approaches but has not yet reached echocardiographic LVEF < 60% or LV end-systolic dimension (ESD) > 40 mm. The uncertainty of this threshold has fueled early surgical intervention for asymptomatic PMR patients (2). As a result of these uncertain guidelines, over 75% of 37,000 PMR patients from 2011 to 2016 present with symptoms or LV dysfunction and only 10% are asymptomatic (6), with the additional caveat that preoperative LVEF < 60% is associated with late mortality (7).

In PMR, LV dimensions and geometry-based volumes belie true LV and left atrial (LA) volumes and LV spherical remodeling obtained with geometry independent cardiac magnetic resonance (CMR) imaging (8, 9). The assessment of PMR is further confounded by a spuriously elevated LVEF due to increased adrenergic drive (10) and ejection into a low-pressure LA. We have shown that even in patients with LVEF > 60%, there is severe cardiomyocyte mitochondrial and cytoskeletal damage, excessive oxidative stress, and interstitial collagen loss, resulting in a decrease in the LV mass/volume ratio and a spherically remodeled LV (11–13).

We recently reported that machine learning models using LVEF, mid LV circumferential strain rate (SR_circ_), LV end-systolic dimension (LVESD), and LV sphericity (SI) predict LVEF < 50% after mitral valve surgery (14). When applying these markers to asymptomatic PMR LVEF > 60% with moderate to severe PMR, 30% of patients were predicted to have post-surgery LVEF < 50% if they had mitral valve surgery (14). The advantages of machine learning models are their ability to integrate predictors extracted from multiple sources and model both linear and nonlinear interactions amongst them (15). The purpose of this study is to identify LV functional and geometric markers that herald a decrease in LVEF < 60% in asymptomatic PMR patients with moderate to severe PMR over 18 months with CMR exams every six months. Using both statistical and machine learning models, we will explore two types of models that employ CMR LVEF, mid LV mid SR_circ_, LVESD, and LV SI features at a single time point (e.g., baseline) and a model utilizing repeated measurements over time.

## Materials and methods

### Study population

This single-center study includes 33 asymptomatic PMR patients recruited between 2006 and 2010 under NHLBI Specialized Centers of Clinically Oriented Research grant (16). Primary degenerative mitral valve prolapse has echocardiographic evidence of thickened, redundant leaflets with excessive motion and prolapse. Patients were excluded for evidence of: (1) aortic valve > trace aortic regurgitation or mean gradient of > 10 mmHg, (2) mitral stenosis (mean gradient > 5 mmHg, valve area < 1.5 cm2), (3) endocarditis, (4) iatrogenic MR (ergot, radiation induced), (5) hemodialysis, (6) pregnancy, (7) presence of coronary artery disease (stenosis > 50%), (8) positive exercise tolerance test with myocardial perfusion. None of the patients were surgical candidates upon entering this study. All patients were asymptomatic and had no history or evidence of coronary artery disease, ruled out by a maximum exercise tolerance test with nuclear imaging. The Institutional Review Boards of the University of Alabama at Birmingham and Auburn University approved the study protocol. All participants gave written informed consent.

All data from patients’ baseline and return visits were obtained prospectively and recorded in electronic health data records. Asymptomatic PMR patients had Class I status, with moderate/severe PMR by color flow Echo/Doppler, LVEF > 60%, LVESD < 40 mm, leaflet thickening and prolapse, and normal maximal exercise myocardial perfusion imaging (16).

### Cardiac magnetic resonance imaging

Magnetic resonance imaging was performed on a 1.5-T MRI scanner (Signa GE, Milwaukee, Wisconsin) optimized for cardiac application. Electrocardiographically gated breath-hold steady-state free precision technique was used to obtain standard (2-, 3-, and 4-chamber short-axis) views using the following parameters: slice thickness of the imaging planes 8 mm, field of view 44 44, scan matrix 256 128, flip angle 45°, repetition/echo times 3.8/1.6 ms. Three-dimensional LV geometric parameters were measured from endocardial and epicardial contours manually traced on cine-MR images acquired near end diastole and end systole. The contours were traced to exclude the papillary muscles. Cubic B-spline surfaces were fit to the endocardial and epicardial contours for each time frame (8–13). The severity of mitral regurgitation (regurgitant volume and regurgitant fraction) was obtained by: Regurgitant volume = LV – RV stroke volume and Regurgitant fraction = LV – RV stroke volume/LV stroke volume.

Tagged magnetic resonance images were acquired with repetition/echo times 8/44 ms, and tag spacing 7 mm (10, 11). Three-dimensional LV strain was measured from tagged images at end systole, which was defined by visual inspection of the image data as the time frame with maximum contraction. Strain computations were conducted using an in-house software package. Two-dimensional strain rates were measured using harmonic phase analysis. Harmonic phase analysis measures the local, 2-dimensional strain of the myocardium based on the local spatial frequency of the tag lines. During myocardial contraction, the tag lines become closer to each other and the tag frequency increases in proportion to that contraction. Strain rates were computed at mid LV segment as defined by Cerqueira et al. (17)

### Calculations

Three-dimensional wall thickness was computed at the same segments by measuring the distance from a point on the endocardial surface to the closest point on the endocardial surface along a line perpendicular to the epicardial surface. The radius to wall thickness ratio was computed as the reciprocal of the product of the endocardial circumferential curvature (*κ*) and wall thickness (*T*). End-systolic wall stress was computed according to the formula (10):

$$\mathrm{Wall}\;\mathrm{stress}=0.133\frac{P}{2kT\left(1+\frac{\kappa T}{2}\right)}$$

where *P* is mean arterial LV blood pressure measured by a cuff measurement at the time of the MR scan. Mean arterial pressure was calculated as: MAP = DP + 1/3(SP – DP) or MAP = DP + 1/3(PP) [systolic blood pressure 2(diastolic pressure)]/3.

### Model development in asymptomatic PMR

The objective of this preliminary study is to develop models for predicting LVEF < 60% in the subsequent (6 month) CMR examination. Due to the limited number of patients, the study employed four features selected from our previous study (14): LVEF, LVESD, LV SI, and mid LV SR_circ_ measured at four time points: baseline, 6, 12, and 18 months. Predictive models were constructed to investigate the following questions: (1) Compared with the model using CMR at a single time point, does the inclusion of features recorded at multiple time points improve the prediction of LVEF < 60%? and (2) What is the optimal number of time points required for accurate prediction in this context?

The final goal of this preliminary study is to develop a predictive model for predicting LVEF < 60% in the subsequent (6 month) CMR examination. Statistical models that model repeated measurements (e.g., mixed-effect model, marginal model) require at least three time points. Thus, we utilized machine learning models to investigate the two questions. After finalizing how many time points to be included in the predictive model, both statistical and machine learning model were fitted for predicting LVEF < 60% at 18 months.

### Random Forest for repeated measures

Since Random Forest (RF) showed superior performance in our previous study (14), we utilized RF to construct three predictive models to investigate the inclusion of features measured at multiple time points. RF is a nonparametric and tree-based approach that operates without assuming a specific distribution of the data. It effectively models complex relationships between variables without assuming a specific function form and is less prone to overfitting especially when dealing with high-dimensional data (18). Three RF models were used to predict whether LVEF is less than 60% at 18 month using CMR features from a) baseline only, b) baseline + 6 months, and c) baseline + 6 month + 12 month. For each model, we performed feature selection and hyperparameter tuning, and assessed the model performance via repeated cross-validation. The metrics used for model assessment include accuracy, sensitivity, specificity, and Area under the ROC curve (AUC) values.

$$Accuracy=\frac{TP+TN}{TP+TN+FP+FN}$$

$$Sensitivity=\frac{TP}{TP+FN}$$

$$Specificity=\frac{TN}{TN+FP}$$

Where *TP, TN, FP*, and *FN* are short for true positive, true negative, false positive, and false negative.

In addition to assessing the performance of the predictive models, the SHapley Additive exPlanations (SHAP) (14, 19) value was used to investigate the importance of each feature and the directional impact of each feature on predicting the drop in LVEF (i.e., a positive or negative impact on LVEF < 60% at 18 months).

### Statistical models

With the four CMR parameters (LVEF, LVESD, LV SI, and mid LV SR_circ_) obtained at baseline, 6 month, and 12 month, we also fitted the Generalized Linear Mixed-effect Model (GLMM) (20) and the Generalized Estimating Equations (GEE) (21) model, which is the marginal model, for the prediction and inference at the patient and population level, respectively. Different from RF models, the response variable in the GLMM and GEE is a length 3 vector, with each element indicating whether LVEF > 60% (coded as 0) or < 60% (coded as 1) at 6, 12, and 18 months and the predictors are the four CMR parameters recorded at baseline, 6, and 12 months.

With GLMM, the effect of the longitudinal features on individual patients is assessed (i.e., subject-level inference). To account for repeated measures within each patient, the GLMM is fitted with a random intercept. The model incorporates follow-up time as a discrete variable and interactions between the features and time to estimate the rate of progression for each feature. The determination of significant predictors and interactions are based on likelihood ratio tests, comparing the coefficient of a predictor being zero vs. non-zero.

The GEE is fitted with a similar model structure and the correlation structure being autoregressive lag 1. Different from GLMM, GEE enables group-level inference, investigating the fixed effects of the longitudinal CMR parameters on a broader population of asymptomatic PMR patients. Due to the scale differences among the four parameters, each parameter was standardized. GLMM and GEE models are assessed by the marginal R squared, which describes the proportion of variance explained by the fixed effects, and conditional R squared, which describes the proportion of variance explained by both the fixed and random effects. GLMM is not applicable to cross validation. Thus, only GEE performance was assessed via repeated cross validation and compared with the RF model. The final model was retrained using all the data, allowing for a comprehensive understanding of the model's coefficients.

### Statistical methods

Data in Table 1 are presented as number/total (%) in group or median with 25% and 75% interquartile range in parentheses.

**Table 1:** Demographics and CMR in asymptomatic PMR patients at baseline.

	PMR (*n* = 33)
Age (years)	53 (45, 62)
Female/male	16 (52%)/15 (48%)
BMI (kg/$m^{2}$)	25.5 (22.0, 27.1)
BSA ($m^{2}$)	1.83 (1.68, 2.06)
LVEF (%)	61.9 (59.0, 67.0)
LVED volume (ml/$m^{2}$)	176.8 (145.6, 204.3)
LVES volume (ml/$m^{2}$)	63.5 (51.5, 78)
LV stroke volume (ml/$m^{2}$)	110.5 (85.8, 129)
LVED diameter (mm)	54 (51.5, 58.5)
LVES diameter (mm)	40.8 (36, 44.7)
LVED mass/volume (g/ml)	0.6 (0.5, 0.7)
LV sphericity index (SI)	1.6 (1.5, 1.7)
LVED radius/wall thickness	4.7 (4.2, 5.4)
LA max volume(ml/$m^{2}$)	42.3 (35.6, 55.9)
LA min volume (ml/$m^{2}$)	20.3 (15.4, 26.9)
Regurgitant volume (ml)	38.6 (26.3, 56.5)
Regurgitant fraction (%)	38.6 (28.7, 51.1)
LV Syst. Circ. strain rate (1/ms)	−0.0007 (−0.0007, −0.0006)
LVES circumferential strain	−0.14 (−0.15, −0.13)
LVES longitudinal strain	−0.14 (−0.16, −0.12)
LVES maximal strain	−0.20 (−0.21, −0.19)

## Results

### Demographics and cardiac magnetic resonance imaging data

Demographics, CMR-derived LV and LA volumes, and LV strains in 33 asymptomatic PMR patients are listed in Table 1.

### Importance of mid LV in PMR

**Figure 1:**
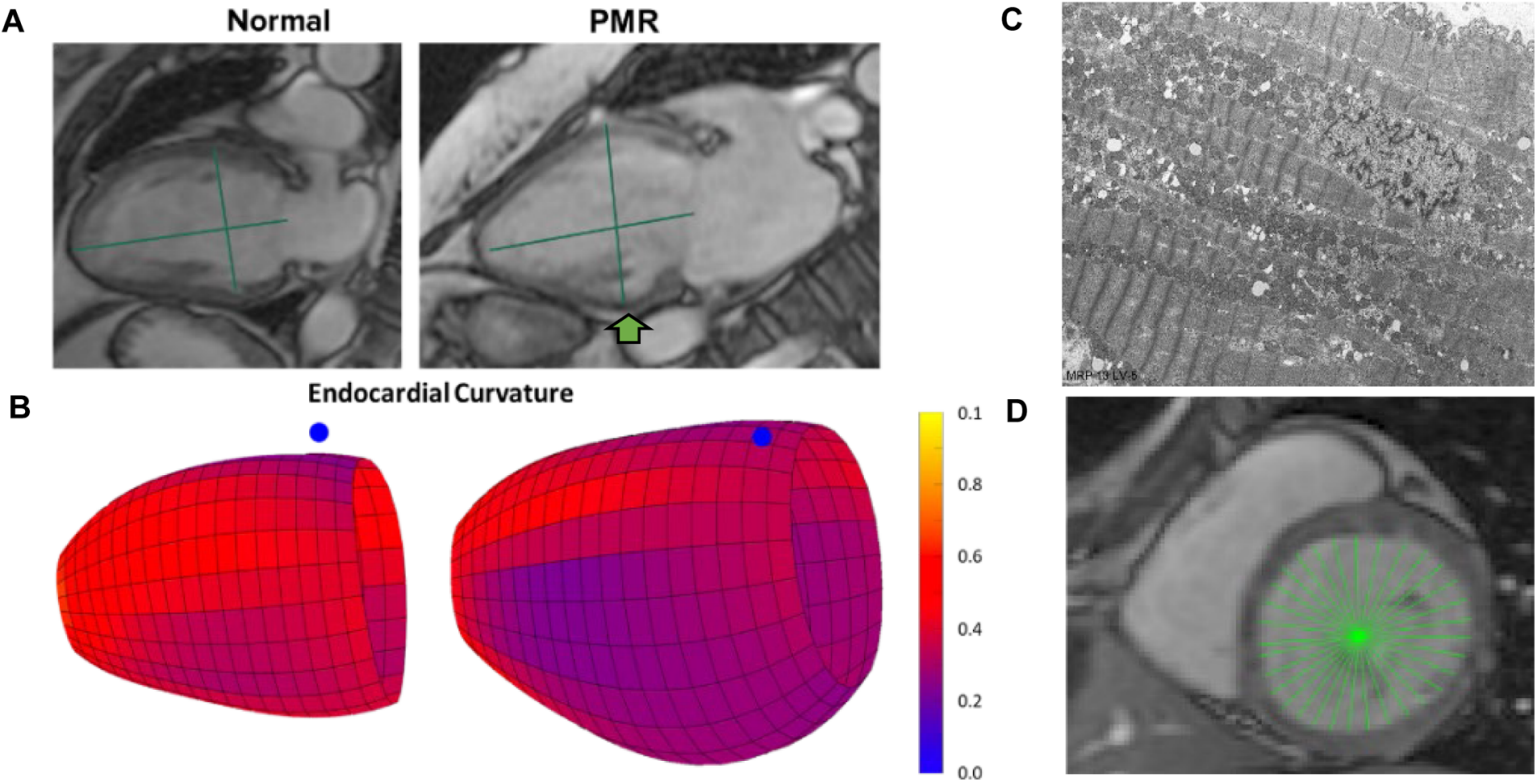
Importance of mid LV remodeling in PMR. **(A)** CMR images of a normal subject and PMR patient demonstrate LV wall thinning and decrease in sphericity index (green lines length/width). **(B)** Color-coded LV maps demonstrating decrease in endocardial curvature (**bluer**) at mid LV (blue dot marks interventricular septum). **(C)** TEM of endomyocardial biopsies from mid LV lateral wall (**green arrow in A**) depicting extensive myofibrillar lysis, and breakdown of sarcomere structure with disorganized and damaged mitochondria. **(D)** CMR LVESD measurement as the sum of 32 radially directed vectors measured at the tips of the papillary muscles in the mid LV near measurement of mid LV circumferential strain rate.

The mid LV is an important point of spherical transition as LV diameter increases to a greater extent than LV length decreasing sphericity index and wall thickness/radius producing an increase in LV wall stress. LV biopsies taken at mid LV from our previous studies (10–12) demonstrate this point in our PMR patients, showing a decrease in LV SI and LV wall thickness (Figure 1A) along with a decrease in mid LV endocardial curvature (Figure 1B). The green arrow in Figure 1A at the location of our myocardial biopsies is coincident with myofibril lysis, sarcomere breakdown, and disorganized mitochondria with cristae lysis (Figure 1C). LVEDD and LVESD (Figure 1D) is the sum of 32 radially directed vectors at mid LV that is close to measurement of mid LV SR_circ_. It is important to note that the location of the myocardial biopsies from our previous studies of PMR patients is in the same location of the CMR derived LVEDD and LVESD (10–12).

### Asymptomatic PMR 18 month time course

**Figure 2:**
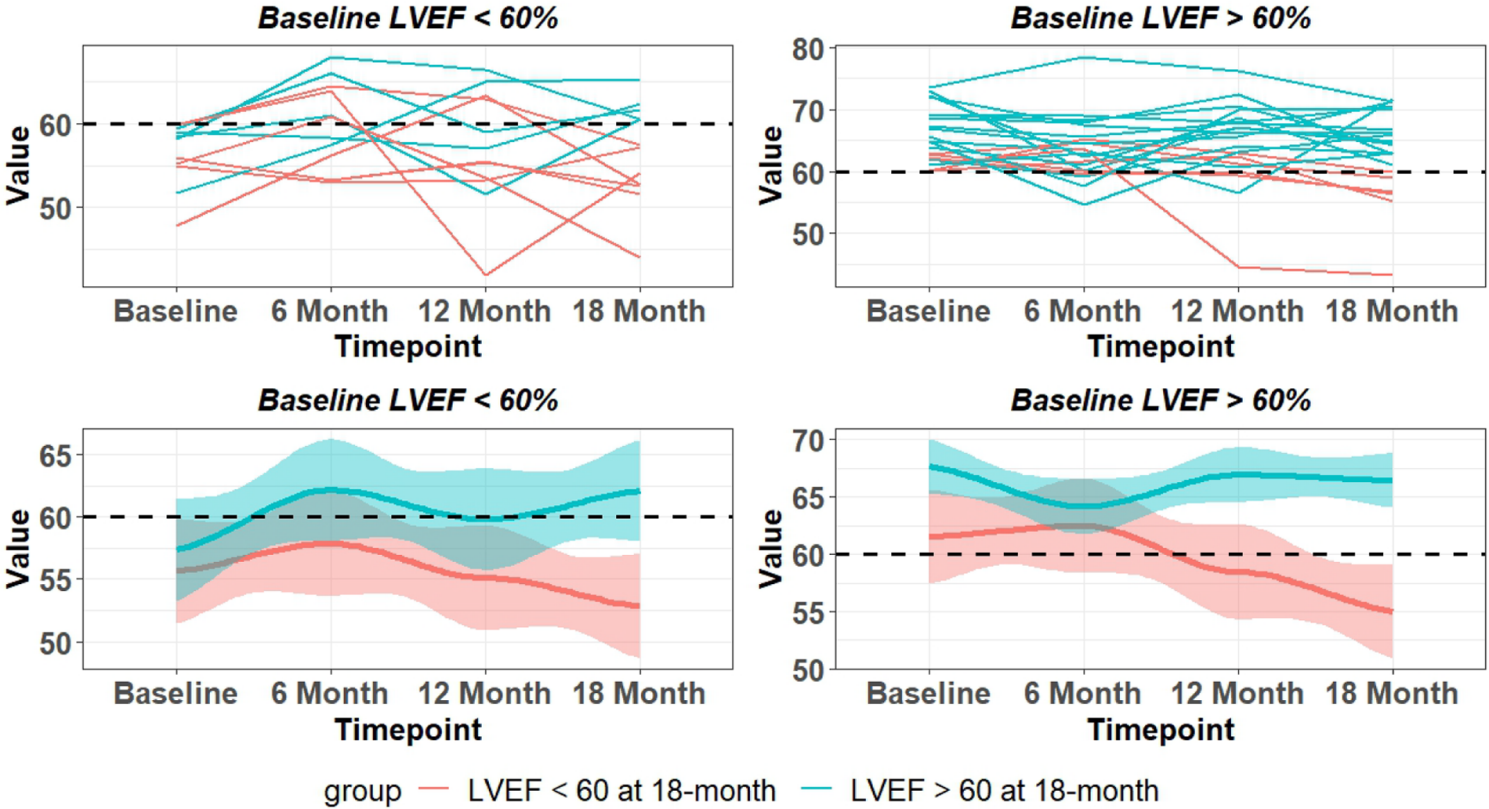
LVEF changes over 18 months in asymptomatic patients with moderate to severe PMR with baseline CMR LVEF < 60% and LVEF > 60%. Top panel are individual patients and bottom panel indicates mean and 95% CI (shaded area). Blue lines – LVEF remains > 60% at 18 months. Red lines LVEF < 60% at 18 months.

The 18-month outcome of 33 PMR patients with CMR baseline LVEF > 60% (*n* = 21) or baseline LVEF < 60% (*n* = 12) is presented in Figure 2. Of 21 patients with baseline LVEF > 60%, 6 patients had LVEF < 60% and 15 had LVEF > 60% at 18 months. Of 12 patients with baseline LVEF < 60%, 5 patients had an increase in LVEF > 60% at 18 months and 7 patients were asymptomatic with LVEF < 60% at 18 months. Of the 33 PMR patients, 13 had CMR LVEF < 60% by 18 months despite all presenting with a median baseline CMR-derived LVEF > 60% (Table 1) and Echo-derived LVEF >60%.

**Figure 3:**
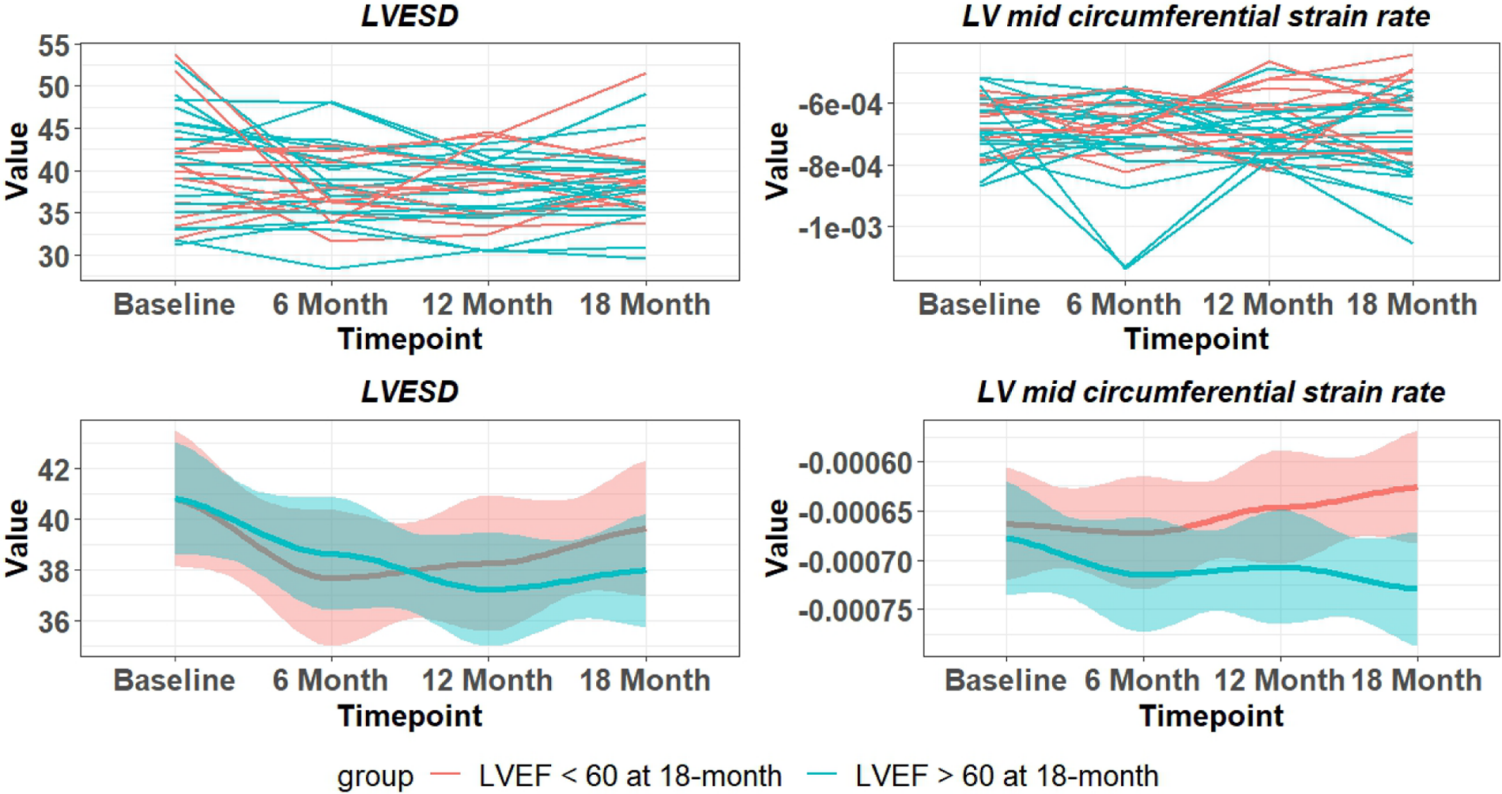
Variability of LVESD and mid LV SR_circ_ over 18 months in PMR patients. Top panel are individual patients and bottom panel indicates mean and 95% CI (shaded area). Blue lines – LVEF remains > 60% at 18 months. Red lines LVEF < 60% at 18 months.

**Figure 4:**
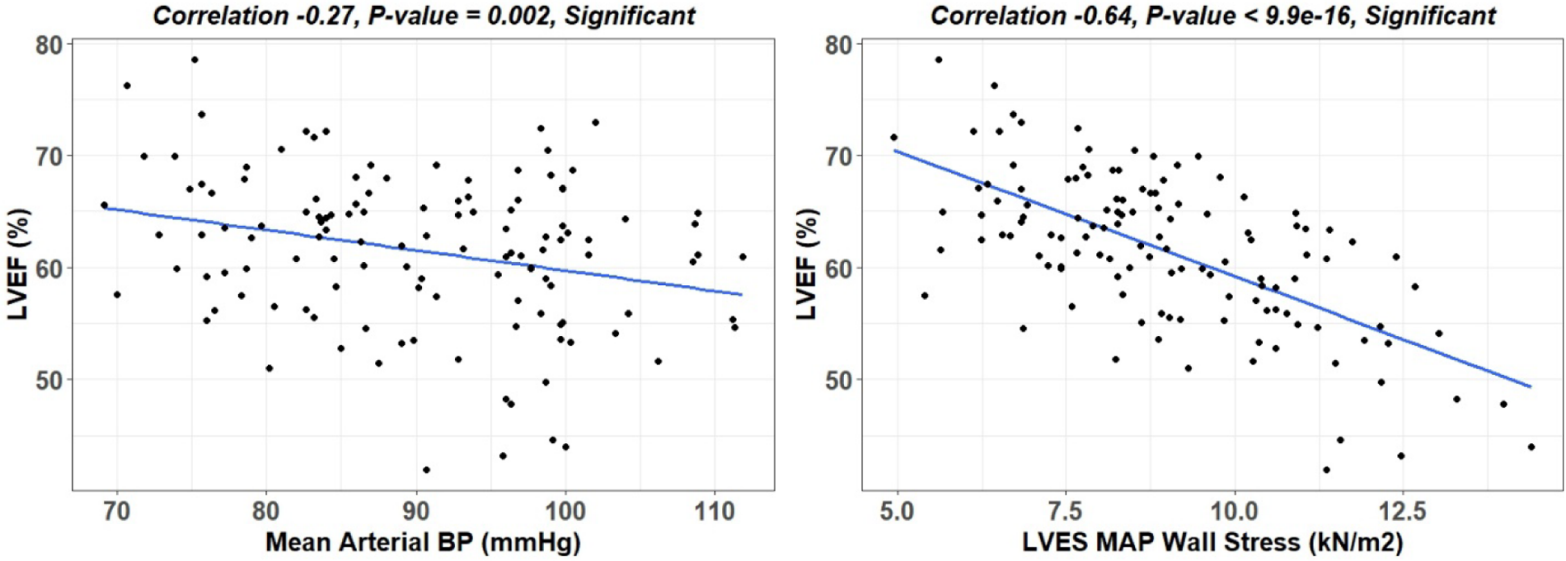
Linear regression of LVEF to mean arterial pressure (MAP) and LVES MAP wall stress. LV end-systolic wall stress provides a more accurate means of defining afterload than MAP, which considers wall thickness and diameter thereby allowing for comparison of different hearts.

There is a wide variability of LVEF, LVESD, and mid LV SR_circ_ at each time point over the 18-month period (Figure 3). These indices of LV shortening are load dependent as demonstrated by the inverse relation of LVEF to mean arterial pressure at the time of imaging and a calculated LV end-systolic wall stress (Figure 4). This demonstrates the inherent physiological variability of LVEF to a changing afterload, militating for repeated measures at the four time points.

### Random Forest models single vs. repeated measures

All patients had four scans (baseline, 6 month, 12 month, and 18 month). To predict the LVEF decline at 18 months, we considered using CMR features measured at baseline only (type 1 model) compared to repeated CMR features measured at two or three time points (type 2 model). In the type 2 model, we considered using baseline + 6 month CMR features and baseline + 6 month + 12 month CMR features respectively. The purpose of constructing these two types of models is to address two questions: (1) if including repeated CMR measurements can improve the prediction of the decline in LVEF at 18 months, compared with a model only using baseline measurements; (2) how many repeated measurements are needed to best predict LVEF decline at 18 months, or in other words, is it necessary to have a CMR scan every six months.

Random Forest models were constructed to predict LVEF < 60% at 18 months. The first model incorporated the four CMR features (LVEF, LVESD, LV SI, and mid LV SR_circ_) identified from our previous study (14) at a single time point (i.e., baseline). The second model integrated the four features measured at two consecutive time points—baseline and 6 months. The third model encompassed the four features measured at baseline, 6, and 12 months. The RF model utilizing the four features at baseline achieved a low prediction accuracy of 64% and sensitivity of 57%, which slightly improved with the second model (72% and 63% respectively) (Table 2). Incorporating the four features (measured at baseline, 6, and 12 months) in the third model achieved highest prediction accuracy (79%) and sensitivity (85%) (Table 2).

### Random Forest vs. statistical models for the prediction of LVEF < 60% at 18 months

Random Forest model was compared to GLMM and GEE statistical models for prediction of LVEF < 60% at 18 months using the same four features measured at baseline, 6, and 12 months. The coefficients of GEE and GLMM are summarized in Table 3. GEE is a marginal model that focuses on estimating the population level effects; while GLMM is the conditional model that focuses on estimating subject-specific effects. For GLMM, the marginal R squared is 0.673 and the conditional R squared is 0.673, and the variance of the subject effect is close to zero. This implies that fixed effects rather than random effects largely explain the drop in LVEF. The GEE marginal R square and conditional R square are 0.736, implying that the GEE population-averaged correlation structure fits the data better and explains more variation in the data providing a better final statistical model for the prediction of LVEF < 60%. A comparison between GEE and RF model performance (using repeated measures of the four features at baseline, 6 months and 12 months) shows that the RF model has a higher prediction accuracy and sensitivity (Table 4).

**Table 3:** Coefficients of GEE and GLM using repeated measures.

Coefficients	GEE	GLM
	Estimate (SE)	Estimate (SE)
Month 6	1.309 (1.09)	1.466 (1.09)
Month 12	1.364 (1.116)	1.548 (1.107)
LVEF	−2.202*** 0.753)	−1.999** 0.894)
LVESD	1.633*** (0.552)	1.518** (0.750)
Sphericity index (SI)	0.103 (0.395)	0.440 (0.678)
LV SR_circ_	−1.502* (0.826)	−1.547* (0.913)
Month 6 × LVEF	0.477 (0.912)	1.022 (1.100)
Month 12 × LVEF	−0.637 (1.52)	0.085 (1.177)
Month 6 × LVESD	−1.538** (0.628)	−1.193 (0.957)
Month 12 × LVESD	−1.858* (0.948)	−1.758* (0.971)
Month 6 × LV SI	−0.771 (0.814)	−1.073 (0.890)
Month 12 × LV SI	0.393 (0.716)	−0.068 (0.787)
Month 6 × LV SR_circ_	2.037* (1.083)	2.608** (1.110)
Month 12 × LV SR_circ_	2.629** (1.137)	2.508** (1.112)

* *p* < 0.1

** *p* < 0.05

**p < 0.01.

Random Forest captures the non-linear relationship between features and the response variables, and further identifies important features that have a strong impact on the LVEF < 60% in a non-linear way. The GEE model assumes a linear relationship between the features and log odds, while RF captures the interactions and combinations among features. Thus, the GEE linear model may not capture such complex relationships; while the data-driven RF model relies heavily on the patterns and information present in the provided data.

### Feature importance in the repeated measures Random Forest model

**Figure 5:**
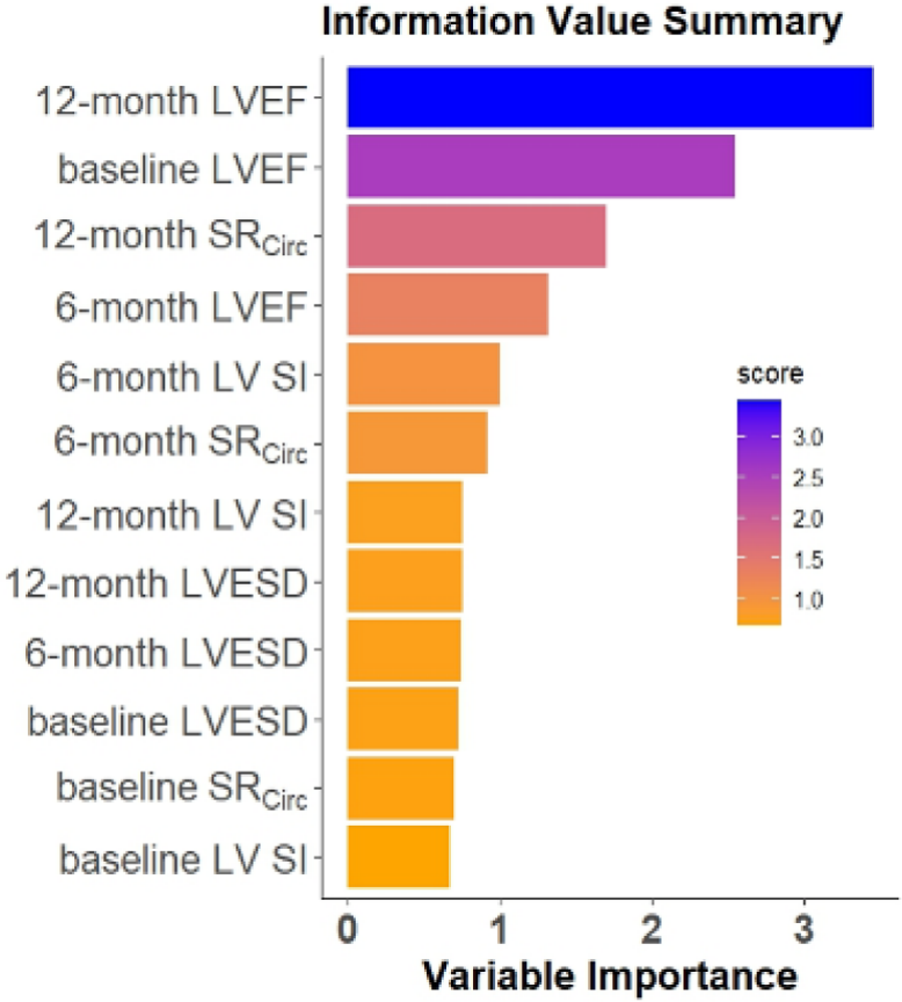
Ranking of feature importance in the repeated measures RF model. The RF model computes the importance of each variable using the four CMR features at baseline, 6, and 12 months (4 features×3 time points) to predict the LVEF < 60% at 18 months.

The RF model computes the importance of each variable using the four CMR features repeatedly measured at baseline, 6, and 12 months (4 features × 3 time points = 12 features) to predict LVEF < 60% at 18 months (Figure 5). The Random Forest model performance used 4 features measured at 3 time points. The RF model utilizing the top 3 features: 12 month LVEF, baseline LVEF, and 12 month mid LV SR_circ_ gave the best model performance in predicting 18 month LVEF < 60% with 79% accuracy and 85% sensitivity (Table 5).

**Table 5:** RF model performance based on 3–12 features in predicting LVEF < 60% at 18 months.

Prediction of LVEF < 60% at 18 months				
Model performance	Total number of features in RF Model			
	3	4	5	12
Accuracy	0.794	0.791	0.766	0.668
Sensitivity	0.850	0.823	0.770	0.565
Specificity	0.753	0.768	0.763	0.746
AUC	0.883	0.886	0.846	0.774

The RF model utilizing the top 3 most important features (Figure 5) has the best model performance.

### Interpretation of Random Forest models

The Shapley Additive exPlanations (SHAP) value (19), inspired by the Shapley value in cooperative game theory, assigns an importance value to each feature in machine learning models to explain the decision made by the model. The SHAP value constructed an overall interpretation of the RF model with the three most important features (12 month LVEF, baseline LVEF, and 12 month mid LV SR_circ_) and its directional impact on prediction (a positive or negative impact on probability of LVEF < 60% at 18 months), and how each feature contributes to a prediction in each patient.

**Figure 6:**
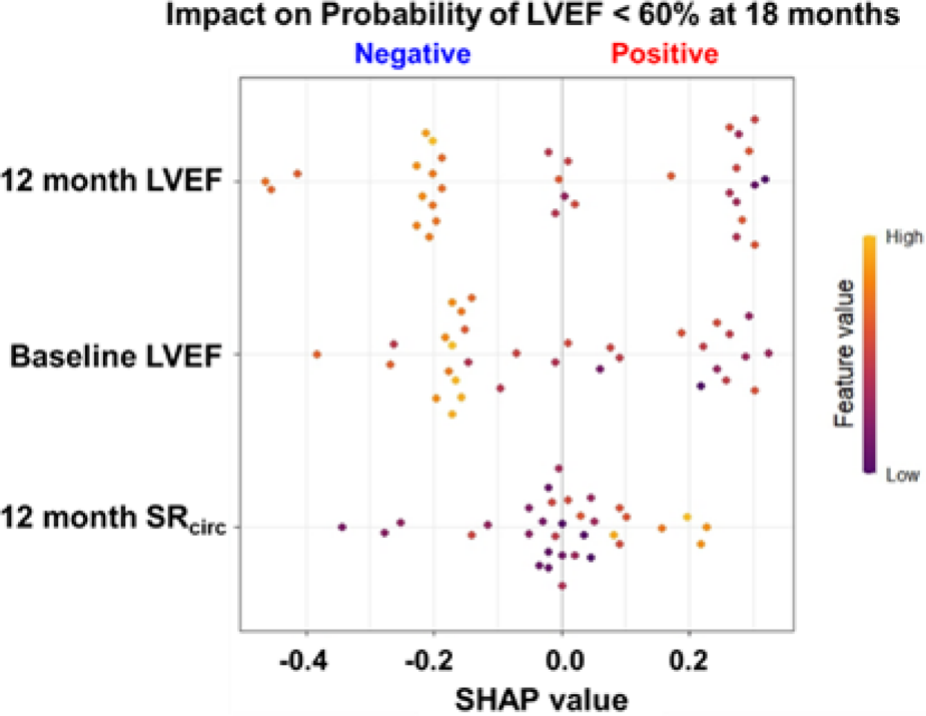
SHAP value for probability for LVEF < 60% at 18 months for the top 3 features in the RF model for asymptomatic PMR. SHAP values quantify the contribution of each feature to model prediction on the *x*-axis. The sign (+/-) of the SHAP value represents the directed impact on probability of LVEF < 60% at 18 months. A positive SHAP indicates high probability while a negative value indicates low probability for LVEF < 60% at 18 months. The magnitude of the SHAP value on the *x*-axis represents the strength of the contribution. Each dot represents an individual patient (*n* = 33), and the color of the dot represents the feature value (color scale), high (orange) and low (purple).

The SHAP value calculated for each patient for the top three features (*y*-axis) is presented in Figure 6. SHAP values quantify the contribution of each feature to model prediction on the *x*-axis. The sign of the SHAP value represents the directed impact on probability of LVEF < 60% at 18 months. A positive SHAP indicates high probability while a negative value indicates low probability for LVEF < 60% at 18 months. The magnitude of the SHAP value on the *x*-axis represents the strength of the contribution. Each dot represents an individual patient, and the color of the dot represents the feature value, high (orange) and low (purple). For baseline and 12 month LVEF, the orange-yellow dots (high LVEF) are located on the left side of the 0 SHAP (i.e., negative SHAP), indicating less probability of LVEF < 60% at 18 months. For 12 month mid LV SR_circ_, the high value (orange-yellow dots) are located on the right side of the 0 (positive SHAP), therefore, a higher 12 month mid LV SR_circ_ (i.e., less negative), the higher chance of developing LVEF < 60% at 18 months.

**Figure 7:**
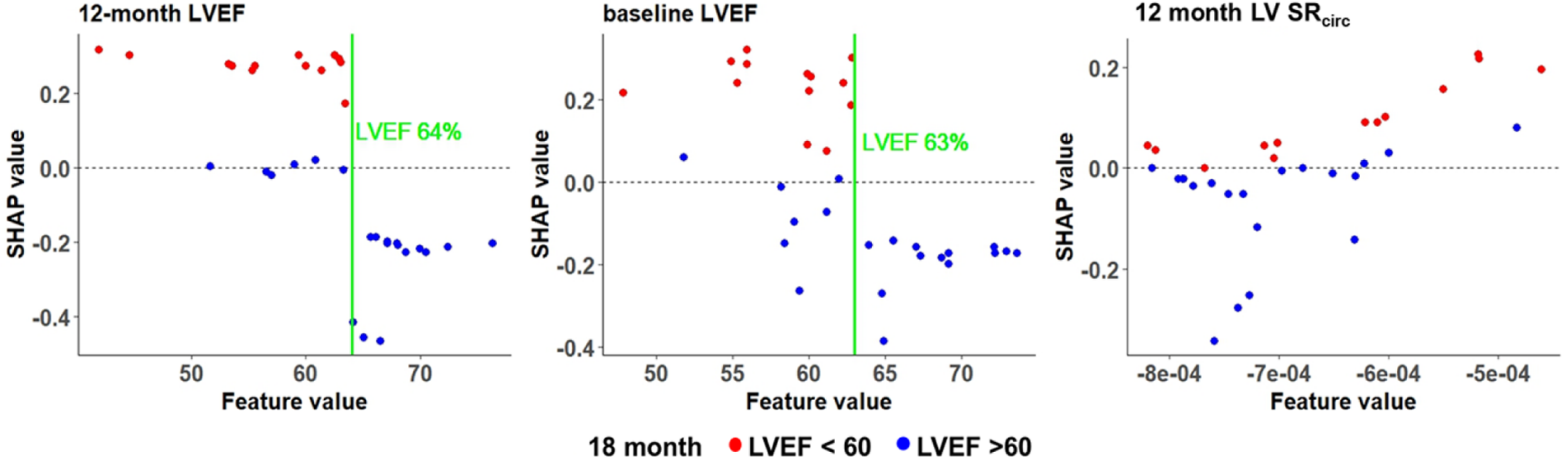
SHAP values for the top 3 features in the RF model for asymptomatic PMR. Manual cutoff values for LVEF (green vertical line) indicate the impact on the likelihood of LVEF < 60% at 18 months. Each dot is an individual patient and colored based on whether the patient has a negative SHAP value indicating lower probability of LVEF < 60% (blue dots) or a positive SHAP value indicating a higher probability of LVEF < 60% (red dots) at 18 months.

To better visualize the top three features (12 month LVEF, baseline LVEF, and 12 month mid LV SR_circ_), the actual value of each feature on the *x* axis and the likelihood of developing LVEF < 60% (red dot) or LVEF > 60% (blue dot) at 18 months is presented in Figure 7. Our data indicates that a higher absolute baseline CMR derived LVEF (> 63%), the less likely for LVEF < 60% at 18 months (negative SHAP values and mostly blue circles). Mid LV SR_circ_ is a negative quantity; thus, more negative values represent a greater LV SR_circ_ and therefore less likely to develop LVEF < 60% at 18 months.

**Table 2:** Random Forest model performance in predicting LVEF < 60% at 18 months comparing single vs. repeated measures over time.

Model Performance	Single	Repeated measures	
	Baseline	Baseline + 6 month	Baseline + 6 month + 12 month
Accuracy	0.64	0.72	0.79
Sensitivity	0.57	0.63	0.85
Specificity	0.69	0.79	0.75
AUC	0.75	0.80	0.88

**Table 4:** Comparison of model performance in predicting LVEF < 60% at 18 months using repeated measures (baseline, 6 months, 12 months).

	GEE	Random Forest
Accuracy	0.65	0.79
Sensitivity	0.50	0.85
Specificity	0.76	0.75
AUC	0.78	0.88

## Discussion

We have previously reported that a combination of statistical methods and machine learning models show that LV SR_circ_, LVESD, LVEF, and LV sphericity index (SI) predict LVEF < 50% after surgery in patients with baseline LVEF > 60% (14). We utilized these same four features to predict the drop in LVEF < 60% over 18 months in 33 asymptomatic patients with moderate to severe PMR with CMR obtained every 6 months. Random Forest models at a single time point (baseline) had a predictive accuracy of 64%. Using repeated measures at baseline, 6, and 12 months achieved a higher predictive accuracy of 79%, improved sensitivity from 57% to 85%, and identified a threshold of CMR LVEF 63%–64% for LVEF < 60%. This pilot longitudinal study in PMR patients provides a stimulus for a longitudinal study that utilizes a more nuanced combination of LV functional parameters that will better inform the clinician of the need for surgery in PMR (including Echo/Doppler derived).

Many studies of PMR have identified predictors of survival or heart failure and death. They include extracellular volume (22–26), regurgitant volume (27), longitudinal strain (28, 29), BNP (30–32), exercise capacity (33), pulmonary artery pressure (34), LA volume (35, 36) and LA emptying fraction (37–39). These studies are largely retrospective, provide just one snapshot in time, and have not addressed the short-term risk for LVEF < 60% in a prospective “***watchful waiting approach” (***40) thus limiting clinical applicability. In the evaluation of asymptomatic PMR patients with LVEF > 60%, it is difficult to know the imminent danger for progression to LVEF < 60%, because surgery in patients with LVEF < 60% has a less favorable outcome. Given the unreliability of LVEF alone, we questioned whether our previous interactive predictors of LV remodeling (LV SI) and LV shortening (LVEF, LVESD, mid LV SR_circ_) features can identify an impending LVEF < 60% in the asymptomatic PMR patient.

The interactive power of machine learning captures the LV spherical remodeling in PMR (8, 9) and its relation to mid LV SR_circ_ affected by the decrease in LV endocardial curvature and LV wall thickness that increases wall stress at the mid LV (Table 1). This is further compounded by severe myofibril lysis, sarcomere breakdown, and disorganized mitochondria with cristae lysis (Figure 1C) ─ all of which contribute to decreased mid LV SR_circ_. In models that determine the effect of LV shape on LVEF, circumferential strain is significantly more important than longitudinal strain in maintaining a normal LVEF in the spherically dilated LV (41). The connection to mid LV SR_circ_ underscores the decrease in contractile velocity that stems not only from myofibril breakdown but also derangement of calcium-handling proteins despite LVEF > 55% in PMR patients (42–45). We have also demonstrated sarcolipin protein upregulation from LV endo-myocardial biopsies in PMR patients (10). Sarcolipin functions as a regulator of SERCA2a by lowering its Ca^2+^ affinity and its inhibitory function is independent of phospholamban (46), both of which control extent and rate of sarcomere shortening.

Based on the coefficients of GEE model in Table 3, there is a predictive variability of LVEF, LVESD, mid LV, SR_circ_ over time. For example, baseline LVEF and 12 month LVEF have a negative while 6 month LVEF has a positive impact. For LVESD, baseline coefficient is positive while 6 and 12 month coefficients are both negative. For mid LV SR_circ_, the baseline coefficient is negative while 6 and 12 month coefficients are both positive. The variability in these indices of LV shortening could be due to the variable blood pressures at each imaging session and also to the small number of patients in this pilot study. This may also explain the higher predictability of three vs. one-time point for predicting LVEF < 60%. LVEF negatively correlated with mean arterial pressure and end-systolic wall stress (Figure 4), which more accurately estimates afterload by incorporating LV radius of curvature and wall thickness. In a stepwise discriminate multivariate analysis of PMR surgery patients, Carabello et al. reported LVES stress/ESV index ratio as the only independent predictor of outcome (47). Future longitudinal studies should include LVES stress/ESV index to normalize effects of afterload on LVEF and other indices of shortening (LVESD and LV mid SRcirc).

The SHAP value identified a CMR LVEF threshold of 63%-64% at baseline, 6 and 12 months. The SHAP value provides an overall interpretation of the machine learning models including a directional positive or negative impact on the probability of LVEF < 60% and a local interpretation at the patient level on how each feature contributes to an individual prediction for each patient. This provides cutoff values that in a larger sample size can comprise a risk score. The SHAP cutoff of 63%-64% is consistent with a study of 300 PMR patients with echo at baseline and within 9-12 months’ post-surgery. The occurrence of post-operative LV dysfunction was 9% when LVEF was ≥ 64% and LVESD < 37 mm and 33% with LVEF < 64% and LVESD ≥ 37 mm (48). Taken together, these results militate for a higher LVEF threshold for mitral valve surgery in PMR.

### Limitations

The obvious limitation of this study is the small number of patients. In this preliminary study, we utilized the classic RF model instead of the Mixed Effects Random Forest (MERF) to model the longitudinal features. Unfortunately, we do not have blood pressure at all time points. In future studies, adding the blood pressure or end-systolic wall stress may provide additive predictive value in normalizing effects of afterload on LVEF. Compared with the classic RF, MERF considers the correlation structure within repeated measurements by incorporating random effects providing accurate predictions for each patient. However, the computational and model complexity of MERF is higher than that of RF due to the inclusion of random effects, which requires more patients. In this preliminary study with the small sample size (*n* = 33) classic RF is preferred over MERF.

## Conclusions

In this pilot study, we identify key LV shortening indices that are widely variable due to the prevailing blood pressures at the time of imaging, which may in part explain the need for more frequent observations. Future studies with a larger number of patients that include blood pressure and end systolic wall stress may improve the predictability of a single study. Taken together, the uncertainty of knowing when the LV approaches but has not yet reached LVEF < 60% calls for a longitudinal study in a larger patient population to test whether a combination of functional features derived from both CMR and Echo/Doppler provides a better indicator for timing of surgical intervention in PMR.

## Data Availability

The raw data supporting the conclusions of this article will be made available by the authors, without undue reservation.
